# Adaptation and Validation of the Child Eating Disorder Examination Questionnaire (ChEDE-Q) for Use in English among Adolescents in Urban India

**DOI:** 10.3390/nu15173836

**Published:** 2023-09-02

**Authors:** Latika Ahuja, Phillippa C. Diedrichs, Kirsty M. Garbett, Anshula Chaudhry, Farheen Hasan, Nora Uglik-Marucha, Silia Vitoratou, Megha Dhillon, Hemal Shroff, Helena Lewis-Smith

**Affiliations:** 1 Centre for Appearance Research, University of the West of England, Bristol BS16 1QY, UK; phillippa.diedrichs@uwe.ac.uk (P.C.D.); kirsty.garbett@uwe.ac.uk (K.M.G.); hasanfarheen95@gmail.com (F.H.); 2 Independent Consultant, New Delhi 110024, India; canshula@outlook.com; 3 Psychometrics and Measurement Lab, Biostatistics and Health Informatics Department, Institute of Psychiatry, Psychology and Neuroscience, King’s College, London SE5 8AF, UK; eleonora.uglik-marucha@kcl.ac.uk (N.U.-M.); silia.vitoratou@kcl.ac.uk (S.V.); 4 Lady Shri Ram College, University of Delhi, New Delhi 110024, India; meghadhillon@gmail.com; 5 Toronto District School Board, Toronto, ON M2N 5N8, Canada; hemal.shroff@gmail.com

**Keywords:** Child Eating Disorder Examination Questionnaire, ChEDE-Q, eating pathology, eating disorders, validation, psychometrics, adolescents, India, Asian, reliability, clinical measure

## Abstract

Eating pathology is increasingly common among Indian adolescents. However, brief validated measures of disordered eating in Indian contexts are scarce. This study adapted and validated a culturally appropriate English language version of the Child Eating Disorder Examination Questionnaire (ChEDE-Q) among 385 adolescents (mean age = 13.42 years; 47.3% girls) in urban India. Confirmatory factor analysis indicated that a two-factor eight-item solution had an acceptable fit to the data across gender: an ‘Eating Concerns and Restraint’ subscale and a ‘Weight and Shape Concerns’ subscale. Further, the questionnaire can be utilised as both a unidimensional and multidimensional tool. This allows for the computation of a total score on the primary factor of ‘Child Eating Pathology’, as well as the two subscales. Internal consistency of the ‘Weight and Shape Concerns’ subscale (α = 0.825) and ‘Eating Concerns and Restraint’ subscale (α = 0.649) was satisfactory. Concurrent validity was established through medium significant correlations with measures of body image and broader mental health. The results support the use of the ChEDE-Q for assessing disordered eating among urban Indian adolescents, thus providing the research community and practitioners with a measure to investigate the nature and scale of disordered eating among adolescents in India.

## 1. Introduction

Disordered eating is a global public health concern [1] and is increasingly common among Indian adolescents [2,3]. Whilst the majority of epidemiological studies on eating pathology have been conducted in Western high-income countries [4,5], several studies show that approximately 26–50% of Indian adolescents, particularly girls, report disordered eating, which increases their risk for threshold eating disorders [6,7]. Further, threshold eating disorders (e.g., anorexia nervosa) are growing among Indian adolescents [8,9,10]. The significant rates of disordered eating in the world’s most populated low–middle-income country warrant concern. Whilst primarily cross-sectional in nature, Indian research indicates severe physical and mental health consequences of disordered eating, including depression and self-harm [2,11,12]. Unsurprisingly, there have been calls from the Indian government to address mental health issues broadly and, particularly, disordered eating [13]. However, prior to intervention development, it is critical to first develop culturally appropriate measures that can accurately and reliably measure the scale of disordered eating in the Indian context [14].

Some of the most frequently used measures of disordered eating in Indian research have included the Sick, Control, One-stone, Fat, Food Questionnaire (SCOFF) [15], the Eating Disorder Inventory (EDI) [16], the Eating Attitude Test (EAT) [17], and the Eating Disorder Examination Questionnaire (EDEQ) [18]. Most of these measures (e.g., SCOFF and EDI), have been used without prior psychometric validation [19]. For example, in a mixed-gender study conducted among Indian university students using the SCOFF, there was no discussion of the instrument’s validation in the Indian context [20]. Although the SCOFF is an effective screening measure with good psychometric properties (Cronbach’s α = 0.73–0.82) [21] in Western contexts, its cultural and psychometric appropriateness for the Indian context remains unexplored. Second, the measures were altered without a clear rationale. For example, one study employed the EAT to examine eating attitudes and body shape concerns among medical students and changed the original Likert response scale (0–3 rating score) into a binary ‘yes’ or ‘no’ format, thus impeding the scale’s ability to sensitively capture variability in eating pathology [22,23]. Third, measures used in Indian research have not undergone a rigorous linguistic translation process, which requires researchers to conduct forward and backward translations, make any necessary changes to the items to enhance cultural and linguistic appropriateness, and conduct acceptability interviews to confirm accurate comprehension of scale items as intended [19].

Overall, this highlights the importance of creating culturally appropriate and psychometrically valid tools to assess the clinical features of disordered eating in India. This is further underscored by long-standing issues regarding the lack of culturally validated mental health assessments in India more broadly [24]. Psychometric validation of measures among adolescents, in particular, is imperative, as this could aid early identification of eating pathology and assist with early intervention and prevention efforts, which are crucial to reducing the burden of eating disorders given mortality rates and reduced quality of life associated with threshold eating disorders [25].

At present, the Eating Disorder Examination Questionnaire (EDE-Q) is the only measure of disordered eating to have undergone rigorous adaptation and psychometric validation among adolescents in the Indian context [23]. The researchers adapted the EDE-Q and assessed the factor structure, reliability, and validity of this measure among 1413 adolescents from urban India (mean age = 13 years; 45% girls). As opposed to the original four-factor model, the study revealed a two-factor model, representing a ‘Preoccupation and Control’ subscale and ‘Weight and Shape Concerns’ subscale for both genders. The adapted scale, comprising 15 items for girls and 18 items for boys, demonstrated satisfactory test–retest reliability and internal consistency (Cronbach’s α = 0.91 for both). This study also reported medium–high correlations between the EDE-Q, measures of body image, and internalisation of appearance ideals, providing evidence for the convergent validity of the measure. Overall, the EDE-Q was indicated as a reliable and valid measure to assess clinical features of eating pathology among adolescents in urban India. However, given the limited resources for mental health in India (e.g., under 1% of the healthcare budget is allocated to mental health) [26], administering such a lengthy measure to assess eating pathology could be problematic. In contrast, the use of the short version of the EDE-Q (i.e., the EDE-Q8) could save time and money and reduce participant burden.

The eight-item EDE-Q (EDE-Q8) is a self-report measure assessing different disordered eating behaviours related to anorexia nervosa (AN), bulimia nervosa (BN), and binge eating disorder (BED) [27]. This version of the EDE-Q has been validated among German adolescents and adults aged 14 to 92 years and provides total and subscale scores indicating the presence or absence of eating pathology. Further, the measure assesses clinical features of eating pathology, including restraint, overeating, food avoidance, preoccupation with food, feelings of fatness, desire to lose weight, guilt about eating, dissatisfaction with weight, and discomfort seeing body. The EDE-Q8 follows a second-order general factor model reflecting restraint, eating concerns, shape concerns, and weight concerns. It has demonstrated good internal consistency reliability (Cronbach’s α = 0.93), and acceptable construct validity, through correlations with the EAT-13 (measure of disordered eating; *r* = 0.75), PHQ-2 (measure of depression; *r* = 0.25), and GAD-2 (measure of anxiety; *r* = 0.27), among non-clinical samples of adults in Europe [28]. After successful validation of the EDE-Q8, this measure was then validated among German children and adolescents aged 7 to 18 years, during which the authors revised its name to the Child EDE-Q (ChEDE-Q) [28]. The measure showed good inter-item correlations (r_it_ > 0.30), reliability (α > 0.90), and model fit for the second-order general factor model, which was similar to EDE-Q8. Construct validity of the ChEDE-Q was established through its correlations with the BESAA (measure of body esteem *r* = 0.65) and the SDQ ‘Emotional Problems’ subscale (a measure of strength and difficulties; *r* = 0.37). This indicates the ChEDE-Q is a reliable and valid measure for assessing clinical features of disordered eating among German adolescents. Further, this suggests that if adapted and validated rigorously, the ChEDE-Q might also be a useful measure of disordered eating behaviours among adolescents in India.

### Present Study

The present study aimed to culturally adapt and examine the factor structure, reliability, and validity of the ChEDE-Q among adolescents in urban India. Given that English is one of the official languages in India [29] and the preferred linguistic medium in urban areas [30], the ChEDE-Q was validated in English. Based on the adaptation and validation of the EDE-Q in the Indian context revealing a two-factor model [23], it was hypothesised that the ChEDE-Q would mirror a similar factor structure, due to its items deriving from the EDE-Q. Concurrent validity of the ChEDE-Q was assessed by correlations with body image (via Body Esteem Scale for Adolescents and Adults (BESAA)) [31], mood (via Positive and Negative Affect Scale (PANAS)) [32], and self-esteem (via Rosenberg Self-Esteem Scale (RSES)) [33]. It was hypothesised that the total and subscale scores of the ChEDE-Q would be negatively correlated with the total and subscales scores of the BESAA, RSES, and positive affect subscale of the PANAS and positively correlated with the negative affect subscale of the PANAS.

## 2. Materials and Method

### 2.1. Participants

This cross-sectional study was conducted between January and April 2019. The measures were completed by 476 adolescents aged 11–17 years (mean age = 13.42, SD = 0.97) across four urban private schools in Delhi and Ludhiana, North West India. This age criterion was determined based on Indian studies that identify early adolescence as a developmentally sensitive period that increases the risk for disordered eating and threshold eating disorders [1].

### 2.2. Measures

#### 2.2.1. Disordered Eating

The Child Eating Disorder Examination Questionnaire (ChEDE-Q) [28] is an 8-item measure (e.g., ‘How unhappy have you been with your weight?’; ‘Have you tried to control the amount of food you eat to change your weight or shape (whether or not you have succeeded)?’) that assesses eating disorders symptomology specifically related to anorexia nervosa, bulimia nervosa, and binge eating disorder. Responses to the items are anchored on a 7-point Likert scale, ranging from 0 (none of the days) to 6 (every day), with higher scores indicating greater disordered eating.

Prior to validating the measure, scale items were adapted for the Indian context. These modifications were made by the eighth and ninth authors, based on their psychological research and cultural expertise (e.g., deletion or modification of particular words). During this process, none of the items were deleted. However, minor linguistic changes were made to simplify the language. For example, the original item *‘Has thinking about food, eating or calories made it very difficult to concentrate on things you were interested in (for example, working, or following a conversation)’* was adapted to *‘On how many of the past 28 days, has thinking about food or calories made it hard for you to pay attention to things you are interested in (for example, watching TV, reading, or playing on the computer)?’*.

Next, the fourth author conducted cognitive interviews with 12 adolescents (50% girls) aged 11–15 years from a private secondary school in Delhi. This sample was balanced across gender, age, and literacy levels. Students were asked to explain each item in their own words. If items were found challenging, the student and interviewee worked collaboratively to agree on an alternative phrasing that best described the scale item. Based on the cognitive interviews, only one scale item was modified. *‘Have you had a strong desire to have an empty stomach with the aim of changing your shape or weight?’* was changed *to ‘Have you had a strong wish to have an empty stomach to try and change your shape or weight?’*.

Measures of body esteem, affect, and self-esteem, were selected to assess concurrent validity based on the existing evidence of associations between eating pathology, low body esteem, negative affect, and low self-esteem in India [23,31,34]. These measures underwent simultaneous psychometric evaluation in India [31,35,36] following vigorous validation guidelines [19], and thus, the items differ from those of the original scales (described below).

#### 2.2.2. Body Esteem

The Body Esteem Scale for Adults and Adolescents (BESAA) [37] is a globally recognised measure of body esteem and has been rigorously validated among adolescents in India [31]. This validation led to two gender-based scales, including a 15-item scale for girls and a 7-item scale for boys. Both scales include Positive Appearance *(e.g., ‘I like what I look like in pictures’)* and Negative Appearance *(e.g., ‘My looks upset me’)* subscales, with the girls’ scale having an additional Weight subscale *(e.g., ‘I am satisfied with my weight’).* The BESAA has demonstrated satisfactory internal consistency (α > 0.70 for girls and α = 0.58 for boys) and good construct validity among Indian adolescents [31]. For this study, both total and subscale scores of the BESAA were used to determine the concurrent validity of the ChEDE-Q.

#### 2.2.3. Affect

The Positive Affect and Negative Affect Scale (PANAS) [32] is a widely recognised measure that has undergone validation among Indian adolescents [36]. Following validation in India, this 10-item measure includes a ‘Positive Affect’ subscale *(‘How often have you felt happy over the past two weeks?’)* and a ‘Negative Affect’ subscale *(‘How often have you felt sad over the past two weeks?’)*. The PANAS has also demonstrated satisfactory internal consistency (α > 0.59 positive subscale, α = 0.70 negative subscale) and good construct validity among Indian adolescents [36]. For this study, both subscale scores were used to determine the concurrent validity of the ChEDE-Q.

#### 2.2.4. Self-Esteem

The Rosenberg Self-Esteem Scale (RSES) [33] is a widely used measure of self-esteem and has been validated among Indian adolescents [35]. This 10-item measure includes a ‘Positive Self-Esteem’ subscale *(‘On the whole I am satisfied with myself’)* and a ‘Negative Self-Esteem’ subscale *(‘At times, I think I am no good at all’)*. The RSES has demonstrated satisfactory internal consistency (α > 0.49 for positive self-esteem, α = 0.67 for negative self-esteem) and good construct validity in the Indian context [35]. For this study, the total score of the RSES was used to determine the concurrent validity of the ChEDE-Q.

### 2.3. Procedure

Ethical approval was sought from the first author’s university ethics committee (HAS.18.01.074). Opportunistic sampling was used to recruit English-medium private secondary schools. Parental or principal consent was sought based on the school’s discretion, as is in accordance with ethical research practice in India. The students were given detailed information sheets concerning the purpose of the study and their role as a participant. They were informed about confidentiality and their rights, including that they could withdraw from the study at any point and that they did not have to answer any questions that made them feel distressed. Next, participants were given the opportunity to clarify their doubts, after which they provided written assent. Questionnaires took approximately 30 min to complete and were administered in classes (<50 students per class). To thank the schools for their participation, schools received INR 13,500 for use toward school equipment.

### 2.4. Analysis

All analyses were conducted using Mplus 8 [38] and SPSS AMOS [39]. Participants with any missing data were removed from the data listwise (*n* = 91), leading to a final sample of 385 adolescents (47.3% girls; 52.7% boys), which was sufficient for the analysis conducted.

#### 2.4.1. Dimensionality

**Figure 1:**
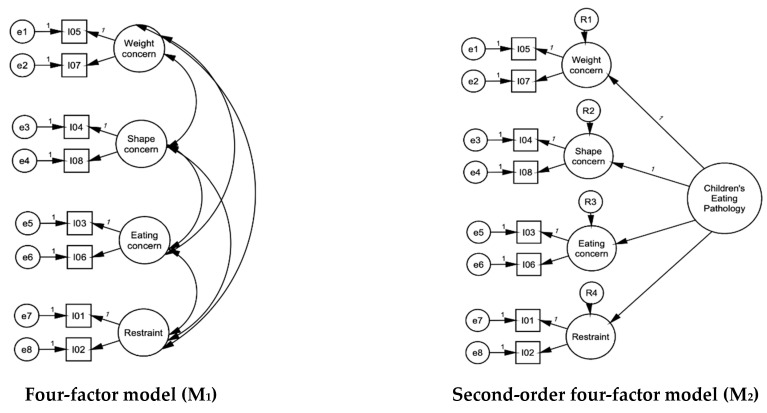

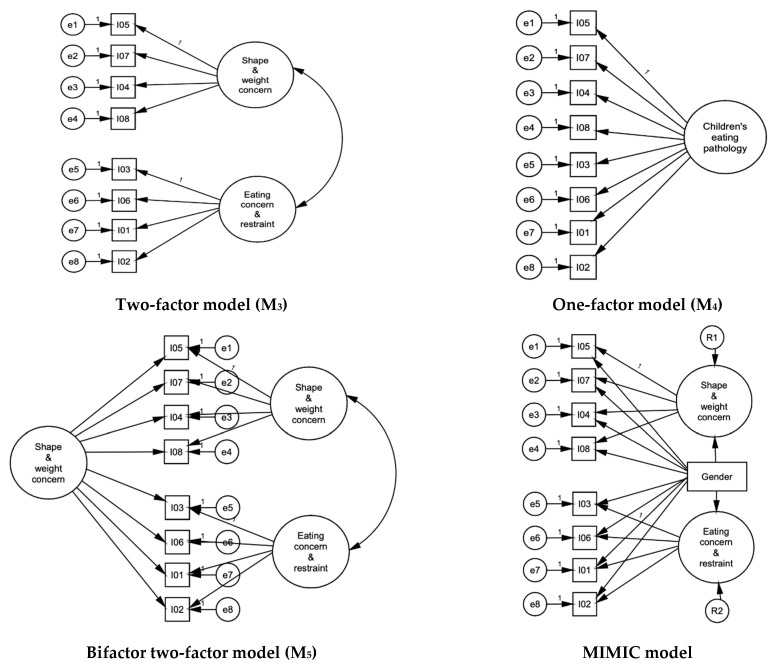
Confirmatory factor analysis model configurations.

The factor structure of the ChEDE-Q was evaluated using confirmatory factor analysis (CFA) via the maximum likelihood estimator (mean-adjusted chi-square test statistic MLM) [40] to account for skewed data. A series of five models were tested: (i) model 1: a four-factor model (‘Weight Concern’, ‘Shape Concern’, ‘Eating Concern’, ‘Restraint’), where each factor comprised two items in line with the hierarchical model (model 2) of Kliem et al. (2015, 2016) [27,28]; (ii) model 2: the Kliem et al. (2015, 2016) [27,28] hierarchical factor model comprising the four factors of model 1, augmented by a second-order factor (‘Children’s Eating Psychopathology’); (iii) model 3: a two-factor model (‘Weight and Shape Concern’ and ‘Eating Concern and Restraint’), where each factor comprised four items; (iv) model 4: a one-factor model, where all eight items loaded on ‘Children’s Eating Psychopathology’ factor; (v) model 5: a bifactor model with two specific factors (‘Weight and Shape Concern’ and ‘Eating Concern and Restraint’) consisting of four items each and a general factor comprising all eight items. See Figure 1 for details.

To test the higher-order factor model (model 2), a single higher-order factor was specified to account for the correlations among the multiple lower-order factors [41]. In the bifactor model (model 5), all items loaded on a general latent factor, which accounted for the commonality of all indicators. In addition to each item being modelled to load on the general factor, items were assigned to specific factors, which were not correlated with the general factor and that could potentially account for item response variance unaccounted for by the general factor [42].

The bifactor model methodology was used to assess whether the ChEDE-Q could be used as a unidimensional measure, multidimensional measure, or both. A procedure outlined by Reise et al. (2007) was followed to evaluate the dimensionality of the ChEDE-Q, by comparing (i) the loadings of the unidimensional model (model 4) versus the loadings of the general factor of the bifactor model (model 5), (ii) the loadings of the two factors in the two-factor model (model 3) versus the corresponding loadings of the specific factors of the bifactor two-factor model (model 5), and (iii) the loadings of the general factor versus the specific factors of the bifactor two-factor model (model 5).

Assessment of model fit using multiple indices is generally recommended to provide different information about the model (for instance, fit relative to a null model, fit relative to a perfect model). Both absolute and relative fits of the models were evaluated using the root mean square error of approximation (RMSEA) [43], the relative chi-square (relative *χ*^2^), the comparative fit index (CFI) [44], the Taylor-Lewis index (TLI) [45], and the standardised root mean residual (SRMR) [43]. Guided by suggestions provided by Hu and Bentler (1999), Bentler (1990), and Bentler and Bonett (1980), a close fit of the model to the data was indicated by RMSEA values below 0.05 (and below 0.08 for acceptable fit), relative *χ*^2^ below 2 (and below 5 for acceptable fit), TLI and CFI values above 0.95, and SRMR values below 0.08. Regarding the assessment of factor loadings, items were considered strongly related to their corresponding factor with values of ≥0.30 (Brown, 2006).

The assessment of measurement invariance (MI) evaluates the extent to which the measure’s psychometric properties are equivalent across different groups. This type of analysis reinforces the investigations of the construct validity of the questionnaire because it offers evidence of construct-irrelevant invariance [46]. The analysis of measurement invariance restricts the development of biased measures and allows for a better interpretation of true score differences underlying the latent constructs [46,47]. Measurement invariance is often tested in the framework of CFA, where the latent factors and items are regressed onto covariates that denote group membership. This approach is known as multiple indicators, multiple causes model (MIMIC) [48,49]. In this study, the gender covariate (girls vs. boys) was added to the model to investigate its direct effects on the items. If the direct effect of gender on a particular item was found as significant, then measurement non-invariance (MNI) with respect to gender was considered evident for that item. MNI denoted that when ‘Children’s Eating Psychopathology’ (latent factor) was held constant, the means of the item were different for girls and boys, leading to gender bias for that item. The analysis was adjusted for age.

#### 2.4.2. Reliability

Cronbach’s alpha (α) [50] and McDonald’s omega (ω) [51] were used as estimates for internal consistency reliability. Alpha is the most prevalent measure of internal consistency despite its very strict assumptions, which are rarely met in the practice of psychological measurement [52]. Less restrictive measures of reliability that incorporate congeneric models are recommended as an alternative to alpha. One such measure is omega, which is the most sensible index of reliability in comparison to both alpha and other alternatives [53,54]. A threshold value of 0.6–0.70 for each coefficient was used to determine satisfactory internal consistency [55]. Reliability was further assessed per item using corrected item-total correlations (ITCs), average inter-item correlations (IICs), and alpha/omega if item deleted (AID/OID). A range of values from 0.2 to 0.8 for ITC indicated satisfactory internal consistency, while items with values of AID/OID above the total alpha/omega were considered problematic.

#### 2.4.3. Validity

The concurrent convergent validity of the ChEDE-Q was assessed through negative correlations (via Spearman’s rho estimates) with the total scores of the BESAA and scores of its negative appearance subscale, similar to the original validation of the ChEDE-Q [27]. The concurrent discriminant validity of the scale was assessed through negative correlations with the positive affect subscale of PANAS. Evidence towards convergent validity was evident through at least moderate correlations with the convergent measure, while discriminant validity was established through weak correlations with the positive affect subscale of PANAS. Concurrent validity was examined through correlations with the RSES.

## 3. Results

### 3.1. Demographic Characteristics

The sample consisted of 385 adolescents (47.3% girls; 52.7% boys), aged 11–17 years (mean age = 13.42, SD = 0.97). All participants were born in India, with the majority (80.8%, *n* = 311) speaking a language other than English at home. Most participants practiced 70.4% (*n* = 217) Islam, followed by Hinduism (28.8%, *n* = 111), Sikhism (0.3%, *n* = 1), Christianity (0.3%, *n* = 1), and other religions (0.3%, *n* = 1). Half of the parents (51.7% mothers, 56.3% fathers) were educated to at least the Bachelor’s degree level.

### 3.2. Factor Structure

CFA was conducted on the entire sample (*n* = 385) with all eight items. Based on the existing literature (Figure 1) [27,28], the four (first-order)-factor model (model 1) was examined first; it included ‘Weight Concerns’, ‘Shape Concerns’, ‘Eating Concerns’, and ‘Restraint’ factors. However, two of these factors (‘Weight Concern’ and ‘Shape Concern’), were collinear, which resulted in an estimated correlation higher than 1 (Heywood case) and led to a non-identifiable model. To address the collinearity, a second-order factor ‘Children’s Eating Pathology’ was added to the model (model 2, Figure 1) following [27,28], which again led to a Heywood case. Negative variance estimations emerged for both factors related to ‘Weight Concern’ and ‘Shape Concern’. To resolve this, these variances were restricted to zero. The restricted model had an acceptable fit to the data (relative $\chi^{2}$ = 2.29, RMSEA = 0.058, CFI = 0.964, TLI = 0.944, SRMR = 0.040). However, there were no theoretical reasons for these restrictions. Thus, multicollinearities were addressed by merging highly correlated factors.

Four items from these two highly correlated factors (‘Weight Concern’ and ‘Shape Concern’) were specified to load on a latent variable of ‘Weight and Shape Concern’. The remaining four items, which assessed food-related concerns and restraint, were merged based on the content of the items and allowed to load on the ‘Eating Concern and Restraint’ factor (model 3; Figure 1). The two-factor solution of model 3 had an acceptable fit to the data (relative $\chi^{2}$ = 2.85, RMSEA = 0.069, CFI = 0.946, TLI = 0.920, SRMR = 0.046), with all items being strongly related to their corresponding latent factors (loadings λ ≥ 0.49; see Table 1).

The unidimensional model (model 4) was also tested, where all eight items loaded on the ‘Children’s Eating Pathology’ factor. The model had an acceptable fit to the data (relative $\chi^{2}$ = 4.06, RMSEA = 0.089, CFI = 0.905, TLI = 0.867, SRMR = 0.059), with the standardised loadings for items being above 0.40. This indicated that all items were meaningful indicators of ‘Children’s Eating Pathology’.

As both model 3 (two-factor model) and model 4 (unidimensional model) demonstrated adequate fit to the data, a corresponding bifactor model (model 5) was also tested, which comprised one general factor (‘Children’s Eating Pathology’) and two specific factors (‘Weight and Shape Concern’, ‘Eating Concern and Restraint’). The standardised factor loadings for all models are displayed in Table 1. Both the standardised loadings of model 4 and the general factor of model 5 were strongly (λ ≥ 0.40) related to their corresponding factors. The presence of specific factors in model 5 did not diminish the strength of the factor loadings of the general factor. Thus, there was no loss of information if the total score of the ChEDE-Q was used. In model 3 (two-factor model) and the specific factors of model 5 (‘Weight and Shape Concern’, ‘Eating Concern and Restraint’), the standardised loadings within each factor were meaningfully (λ ≥ 0.31 except for I03 of model 5) linked with their specified factors. Although in the presence of the general factor in model 5, the loadings of the specific factors (‘Weight and Shape Concern’, ‘Eating Concern and Restraint’) diminished in magnitude when compared to model 3 (two-factor model), they still remained strong, which provided support for the robustness of two-factor structure. In model 5, the loadings of the specific factors remained strong (λ ≥ 0.31) in the presence of the general factor, except for I03 (related to ‘Preoccupation with food’) and I06 (related to ‘Guilt about eating’), whose loadings were non-significant. This indicated that these two items were more strongly related to the general factor than the factor of ‘Eating Concern and Restraint’.

Given that most items were meaningfully related to their specific factors, the use of a two-factor model for the concept of children’s eating psychopathology was supported. Further, considering that the loadings of the general factor were strong, this indicates that the ChEDE-Q can also be used to yield a total score on the primary latent variable measured by the scale (‘Children’s Eating Pathology’), as well as the specific factors measured by the ‘Eating Concern and Restraint’ and ‘Weight and Shape Concern’ subscales.

### 3.3. Measurement Invariance

The MIMIC model was used to assess the invariance of the ChEDE-Q with respect to gender adjusted for age. The non-invariance (bias) with respect to gender is presumed for an item if the direct effect of gender on that particular item is significant. A small significant direct effect of gender was observed for item I07 (de = 0.439; ‘Dissatisfaction with weight’) adjusted for age. Girls were more likely to endorse this item as they scored 0.439 units higher on I07 (on a scale of 0 to 6) than boys. The measurement invariance was deemed negligible given that only one item out of eight was invariant, indicating that the total score between girls and boys was comparable. No differences in the ChEDE-Q scores were found between girls and boys (see Table 2).

### 3.4. Reliability

The two-factor model had satisfactory internal consistency for each subscale as demonstrated by both Cronbach’s alpha (α = 0.65–0.83) and McDonald’s omega (*ω* = 0.64–0.83). Additional internal consistency explorations regarding average IIC, ITC, AID, and OID showed no problematic items (see Table 3).

### 3.5. Concurrent Validity

Weak to moderate correlations emerged between the ChEDE-Q and its subscales and the concurrent measures (except for the positive affect subscale of PANAS; see Table 3). Convergent validity was evident through moderate correlations with the total score of the BESAA and its negative appearance subscale. No associations were found with the positive affect subscale of the PANAS, pointing towards evidence of the discriminant validity of the ChEDE-Q.

## 4. Discussion

The present study aimed to examine the factor structure, reliability, and construct validity of a culturally adapted English version of the ChEDE-Q among adolescents in urban India. An eight-item measure demonstrated psychometrically sound properties and gender-related measurement invariance. Consequently, the ChEDE-Q is a reliable and valid measure for assessing eating psychopathology among urban Indian adolescents.

Unlike the original validation of the ChEDE-Q, which proposed a hierarchical factor model comprising four factors [28], the present analyses revealed a two-factor solution, comprising ‘Eating Concern and Restraint’ and ‘Weight and Shape Concern’ subscales, with four items per subscale. These findings are similar to the validation of the (longer) EDE-Q among Indian adolescents, which also revealed two similar subscales [23], suggesting that these two constructs hold among Indian adolescents even when the number of items is reduced. This is supported by other validations of the EDE-Q among adolescents in non-Western cultures, including in Mexico and Fiji [56,57], where the original subscales were also collapsed. This highlights the potential for cultural differences in the presentation of eating pathology, particularly between Western and non-Western cultures.

The present findings suggest that Indian adolescents may not differentiate between restraint and eating concerns, which could relate to their age [58], whereby clinical features may cluster together and only manifest as distinct subtypes (e.g., anorexia nervosa, bulimia nervosa) later in adolescence [59]. Indeed, the lack of differentiation between restraint and eating concerns among the present sample may be attributed to India’s collectivist culture, where there is less individual external control. For example, meals tend to be eaten as a family, with staff preparing meals in higher socioeconomic groups, meaning some adolescents have minimal control over what they eat. Also, adolescents are expected to eat all of what is given to them during family mealtimes, thus reducing individual control over the amount they eat. Similarly, the collapsing of two original ChEDE-Q subscales into ‘Weight and Shape Concerns’ in the present study indicates that Indian adolescents may perceive weight and shape as one construct [23]. This reflects theories of body image that define body dissatisfaction as a unified construct, capturing both weight and shape concerns [60].

Unlike previous validations of the EDE-Q [61,62], including among Indian adolescents [23], no items were dropped from the ChEDE-Q among the present sample. These findings suggest that the ChEDE-Q captures culturally appropriate disordered eating attitudes and behaviours among this group. A small direct effect of gender was observed on only one item, whereby girls were more likely to endorse ‘Dissatisfaction with weight’. However, this is unsurprising, as girls experience greater levels of weight concerns and body dissatisfaction compared to boys and thus experience higher disordered eating in India [2,3,63]. Nonetheless, the full eight-item ChEDE-Q can be used comparatively to assess disordered eating across genders in urban India.

With regard to construct validity, the ChEDE-Q showed moderate negative correlations with the BESAA. Whilst the ChEDE-Q has not been validated widely, these findings support previous validation studies of the EDE-Q among adolescents [56,57,64], including among Indian adolescents [23]. The ‘Weight and shape’ subscale of ChEDE-Q was more strongly correlated with the BESAA compared to the ‘Eating Concern and Restraint’ subscale. This is not surprising, as weight and shape dissatisfaction is prevalent in urban India [65,66], and the items of the ‘Weight and Shape’ subscales of ChEDE-Q and BESAA are similar [23]. The ChEDE-Q also showed a medium positive correlation with negative mood among adolescents who participated in this study, which adds to the growing body of evidence suggesting an association between depressive mood and disordered eating among this group [67]. This association can also be explained by the distressing nature of eating psychopathology, which is often associated with negative mood among Indian adolescents [68]. Lastly, there was a moderate negative correlation between the RSES and the ChEDE-Q in the expected direction, adding to the evidence that low self-esteem is associated with eating psychopathology among adolescents in India [12,69], as well as globally [70]. Collectively, these findings indicate that the ChEDE-Q is a psychometrically sound measure that can be used to appropriately measure clinical features of eating disorders in India.

Despite the study’s contributions, it is not without its limitations. First, this measure was adapted and validated among adolescents in urban Delhi and Ludhiana, who studied in private schools. These schools were recruited via opportunistic sampling. Given that the education systems are highly varied in private versus public schools in urban India [71], the applicability of these findings is limited. Thus, we recommend that future research evaluates the applicability of this measure at a larger scale across different private and public schools in urban India. Second, English is primarily spoken in urban India. Given that much of the Indian population lives in rural parts where Hindi or other regional languages are more widely spoken instead of English, the usability of the English version of the ChEDE-Q is likely to be restricted to urban samples. However, we are also currently translating and validating the ChEDE-Q in Hindi to ensure the applicability and appropriateness of this measure for adolescents in rural India. Third, it was not feasible to examine the test–retest reliability of the ChEDE-Q. However, test–retest reliability of the EDE-Q has been confirmed among Indian adolescents [23]. Thus, future research needs to evaluate the test–retest reliability of this measure. Lastly, it should be noted that the data were collected in 2019 and thus might not fully reflect adolescents’ disordered eating today in urban India. Thus, further research using this measure is encouraged.

Nonetheless, the study is associated with strengths. First, the ChEDE-Q is the second psychometrically sound measure that can be used to screen eating pathology among adolescents in urban India, providing a more time-sensitive option compared to the EDE-Q. Further, its rigorous adaptation highlights its cultural sensitivity, and its short length endorses its cost-effectiveness. Regarding implications, this study provides evidence that the ChEDE-Q is a psychometrically sound measure that can generate separate scores relating to ‘Shape and Weight Concern’ and ‘Eating Concern and Restraint’, as well as a single overall score of eating pathology. Thus, this measure will allow clinicians to detect young people in India who are at high risk of developing eating disorders and assist researchers in determining the nature and scale of eating pathology in the Indian context. This will help respond to calls from the Indian government to address mental health issues [13]. Further, the short length of this measure is conducive to time and cost restraints in the Indian mental health system [26] and will be more acceptable to adolescents than longer measures assessing disordered eating [28].

These findings support the ongoing argument that there are cultural differences in the manifestation of eating disorders, which is indicated by the two-factor solution. However, given that eating disorder research is still limited in India, future studies could use the ChEDE-Q to understand whether this measure accurately captures the presentation of eating pathology in this cultural context. Finally, whilst the present study provides evidence that the ChEDE-Q is a reliable measure to assess eating pathology in non-clinical samples, this measure needs to undergo validation in clinical samples to examine whether the two-factor structure model holds equally across groups.

## 5. Conclusions

This is the first study to validate a brief measure to assess the clinical features of disordered eating among Indian adolescents. Findings revealed a psychometrically sound, eight-item, two-factor structure of the ChEDE-Q that can be used comparatively across genders to assess eating pathology among adolescents in urban India. It is hoped that the validated measure will encourage further research on the nature and scale of eating pathology among adolescents in India.

## Figures and Tables

**Table 1:** Standardised CFA loadings for 1-factor, 2-factor, and bifactor 2-factor models of the ChEDE-Q and descriptive indices per item (*n* = 385).

Item	Label	Mean (SD)	Median (Min–Max)	1-Factor Model (M_4_)	2-Factor Model (M_3_)		Bifactor 2-Factor Model (M_5_)		
				General Factor	Eating Concern and Restraint	Weight and Shape Concerns	General Factor	Eating Concern and Restraint	Weight and Shape Concerns
I02	Avoidance of eating	0.94 (1.46)	0 (0–6)	0.432	0.529		0.545	0.39	
I01	Food avoidance	1.24 (1.72)	0 (0–6)	0.486	0.6		0.612	0.313	
I06	Guilt about eating	0.98 (1.53)	0 (0–6)	0.571	0.636		0.776	−0.456 *	
I03	Preoccupation with food	1.46 (1.92)	1 (0–6)	0.396	0.491		0.473	−0.038 *	
I04	Feelings of fatness	1.13 (1.82)	0 (0–6)	0.824		0.836	0.592		0.589
I07	Dissatisfaction with weight	1.38 (1.81)	1 (0–6)	0.675		0.685	0.443		0.538
I05	Desire to lose weight	1.35 (2.07)	0 (0–6)	0.812		0.823	0.581		0.584
I08	Discomfort seeing body	1.47 (1.85)	1 (0–6)	0.6		0.593	0.505		0.324

Note. SD: standard deviation, * values are not significant, *p* > 0.05.

**Table 2:** Descriptive indices for all scales and correlations (Spearman’s rho) of the ChEDE-Q with concurrent measures.

	Mean (SD)	Girls (*n* = 182)	Boys (*n* = 203)	Comparison		Total	Eating Concerns and Restraint	Weight and Shape Concerns
		Mean (SD)	Mean (SD)	Statistic	*p*-Value			
Child Eating Disorder Examination Questionnaire (ChEDE-Q; *n* = 385)								
Total	9.96 (9.53)	10.92 (10.88)	9.56 (9.01)	U = 10,277.00	0.604	1		
Eating concerns and restraint	4.63 (4.65)	4.53 (4.9)	4.8 (4.85)	U = 9581.500	0.615	0.872 **	1	
Weight and shape concerns	5.33 (6.12)	6.39 (6.94)	4.76 (5.41)	U = 9581.500	0.117	0.881 **	0.569 **	1
Positive and Negative Affect Scale (PANAS; *n* = 282)								
Positive affect	18.54 (4.39)	18.58 (4.07)	18.51 (4.69)	U = 9791.00	0.847	−0.07	−0.054	−0.078
Negative affect	11.18 (3.74)	11.57 (3.64)	10.83 (3.8)	t(280) = −1.668	0.096	0.307 **	0.294 **	0.264 **
Body Esteem Scale (BES; *n* = 282)								
Total	3.79 (0.75)	3.84 (0.79)	3.75 (0.70)	t(280) = −1.091	0.276	−0.434 **	−0.360 **	−0.421 **
Negative appearance	4.17 (0.76)	4.18 (0.78)	4.17 (0.75)	U = 10,032.000	0.871	−0.463 **	−0.360 **	−0.473 **
Positive appearance	3.29 (1.11)	3.4 (1.16)	3.19 (1.06)	t(280) = −1.600	0.111	−0.290 **	−0.254 **	−0.266 **
Rosenberg Self-Esteem Scale (RSES; *n* = 282)								
Total	20.26 (4.32)	20.12 (4.36)	20.38 (4.3)	t(280) = 0.509	0.611	−0.307 **	−0.297 **	−0.267 **

Note. SD: standard deviation, U: Mann–Whitney test, t: independent samples *t*-test, ** *p* < 0.001.

**Table 3:** Internal consistency indices of the ChEDE-Q within each factor (*n* = 385).

Item	Label	Average IIC	ITC	AID	OID
Weight and shape concerns (α = 0.825, ω = 0.825)					
I04	Feelings of fatness	0.51	0.71	0.75	0.76
I07	Dissatisfaction with weight	0.54	0.66	0.78	0.79
I05	Desire to lose weight	0.52	0.69	0.76	0.76
I08	Discomfort seeing body	0.61	0.55	0.82	0.83
Eating concern and restraint (α = 0.649, ω = 0.637)					
I01	Food avoidance	0.29	0.47	0.551	0.57
I02	Avoidance of eating	0.33	0.425	0.587	0.59
I03	Preoccupation with food	0.34	0.398	0.612	0.64
I06	Guilt about eating	0.32	0.441	0.574	0.59

Note. IIC: average inter-item correlation; ITC: item-total correlation; AID: alpha if item deleted; OID: omega if item deleted; ω: McDonald’s omega; α: Cronbach’s alpha.

## Data Availability

The data presented in this article are not publicly available.
